# Store-Operated Calcium Channels Are Involved in Spontaneous Slow Calcium Oscillations in Striatal Neurons

**DOI:** 10.3389/fncel.2019.00547

**Published:** 2019-12-17

**Authors:** Satomi Kikuta, Yoshio Iguchi, Toshikazu Kakizaki, Kazuto Kobayashi, Yuchio Yanagawa, Masahiko Takada, Makoto Osanai

**Affiliations:** ^1^Department of Radiological Imaging and Informatics, Tohoku University Graduate School of Medicine, Sendai, Japan; ^2^Systems Neuroscience Section, Primate Research Institute, Kyoto University, Inuyama, Japan; ^3^Research Fellow of Japan Society for the Promotion of Science, Tokyo, Japan; ^4^Department of Molecular Genetics, Institute of Biomedical Sciences, Fukushima Medical University School of Medicine, Fukushima, Japan; ^5^Department of Genetic and Behavioral Neuroscience, Gunma University Graduate School of Medicine, Maebashi, Japan; ^6^Laboratory for Physiological Functional Imaging, Department of Medical Physics and Engineering, Division of Health Sciences, Osaka University Graduate School of Medicine, Suita, Japan

**Keywords:** store-operated calcium entry, basal ganglia, striatum, calcium oscillations, fluorescence microscopy

## Abstract

The striatum plays an important role in linking cortical activity to basal ganglia output. Striatal neurons exhibit spontaneous slow Ca^2+^ oscillations that result from Ca^2+^ release from the endoplasmic reticulum (ER) induced by the mGluR5-IP3R signaling cascade. The maximum duration of a single oscillatory event is about 300 s. A major question arises as to how such a long-duration Ca^2+^ elevation is maintained. Store-operated calcium channels (SOCCs) are one of the calcium (Ca^2+^)-permeable ion channels. SOCCs are opened by activating the metabotropic glutamate receptor type 5 and inositol 1,4,5-trisphosphate receptor (mGluR5-IP3R) signal transduction cascade and are related to the pathophysiology of several neurological disorders. However, the functions of SOCCs in striatal neurons remain unclear. Here, we show that SOCCs exert a functional role in striatal GABAergic neurons. Depletion of calcium stores from the ER induced large, sustained calcium entry that was blocked by SKF96365, an inhibitor of SOCCs. Moreover, the application of SKF96365 greatly reduced the frequency of slow Ca^2+^ oscillations. The present results indicate that SOCCs contribute to Ca^2+^ signaling in striatal GABAergic neurons, including medium spiny projection neurons (MSNs) and GABAergic interneurons, through elevated Ca^2+^ due to spontaneous slow Ca^2+^ oscillations.

## Introduction

The calcium ion (Ca^2+^) is an important messenger for signal transduction, and intracellular Ca^2+^ concentration ([Ca^2+^]_i_) changes in response to various physiological stimuli in both excitable and non-excitable cells (Pasti et al., 1997; Smetters et al., 1999; Berridge et al., 2000). Intracellular Ca^2+^ can modulate the functions of proteins such as enzymes and receptors, gene expressions, and morphological changes in cellular processes. The endoplasmic reticulum (ER) is a source of [Ca^2+^]_i_ and is crucial for second messenger-induced intracellular Ca^2+^ signaling (Blaustein and Golovina, 2001; Berridge, 2002). Therefore, Ca^2+^-release from the ER contributes to the modulation of neuronal signal processing in the central nervous system (Kostyuk and Verkhratsky, 1994). In the basal ganglia, the striatum receives inputs from the cortex and is thought to play crucial roles in controlling somatic motor movements, behavioral patterns, cognition, learning, and memory (Graybiel, 1995; Chesselet and Delfs, 1996). However, the cellular Ca^2+^ signaling in striatal neurons still remains unclear. We previously reported long-lasting spontaneous intracellular Ca^2+^ oscillations in rodent striatal neurons (Osanai et al., 2006; Tamura et al., 2014), which lasted as long as 300 s. These slow Ca^2+^ oscillations were not induced by action potentials, but by Ca^2+^ release from the ER. Although both the metabotropic glutamate receptor type 5 and inositol 1,4,5-trisphosphate receptor (mGluR5-IP3R) signal transduction cascade were involved in these slow Ca^2+^ oscillations (Tamura et al., 2014), an important issue arises as to how such a long elevation in Ca^2+^ is maintained.

Store-operated calcium channels (SOCCs) are one of the Ca^2+^ -permeable plasmalemmal ion channels through which Ca^2+^ flows into the cell from the extracellular space when ER stores are depleted (Brini et al., 2014; Majewski and Kuznicki, 2015; Wegierski and Kuznicki, 2018). Although SOCCs have been most thoroughly studied in non-neuronal cells and knowledge of their function in the brain is still poor, data concerning their physiological and pathological functions in neuronal cells are accumulating (Baba et al., 2003; Bouron et al., 2004; Shideman et al., 2009; Zhang et al., 2015; Secondo et al., 2018; Wegierski and Kuznicki, 2018; Ong et al., 2019).

The very long-lasting [Ca^2+^]_i_ elevations that we observed in the form of spontaneous slow Ca^2+^ oscillations in the striatum (Osanai et al., 2006; Tamura et al., 2014) led us to hypothesize that Ca^2+^ in the ER may be depleted and that SOCCs may then be opened when the slow Ca^2+^ oscillations occur. This opening of SOCCs may be involved in the mechanism of maintaining high [Ca^2+^]_i_ for a long time in striatal neurons exhibiting these slow Ca^2+^ oscillations. The present study was designed to examine this hypothesis.

## Materials and Methods

### Mice

In the Ca^2+^ imaging study, to discriminate striatal GABAergic neurons, we used heterozygous GAD67-GFP knock-in mice (GAD67-GFP mice), in which enhanced GFP is selectively expressed under the control of the endogenous GAD67 gene promoter (Tamamaki et al., 2003). The colony was maintained by crossing male GAD67-GFP mice with female C57BL/6 mice (Clea Japan). All mice were housed and maintained at 22–24°C on a 12-h light/ dark cycle and permitted *ad libitum* access to food and water. A total of 10 mice were used in the studies.

### Ca^2+^ Imaging

The methods for Ca^2+^ imaging were described previously (Osanai et al., 2006; Tamura et al., 2014; Kikuta et al., 2015). Briefly, postnatal day 12 (P12) to P17 GAD67-GFP mice of either sex were anesthetized with isoflurane (Mylan) and decapitated. The brain was rapidly isolated and placed in ice-cold artificial cerebrospinal fluid (ACSF) bubbled with 95% O_2_–5% CO_2_. The composition of normal ACSF was as follows (in mM): 137 NaCl, 2.5 KCl, 0.58 NaH_2_PO_4_, 1.2 MgCl_2_, 2.5 CaCl_2_, 21 NaHCO_3_, and 10 glucose. Ca^2+^-free ACSF was made by omitting CaCl_2_ and adding 7.5 mM NaCl. Corticostriatal sagittal slices (300 μm thick) were prepared using a vibratome tissue slicer (VT-1200S, Leica Microsystems) and incubated at room temperature in a submerged chamber containing gassed ACSF for at least 60 min prior to the Ca^2+^-sensitive Fura-2 LR/AM (Calbiochem) fluorescent dye, loading.

As previously described (Kikuta et al., 2015), [Ca^2+^]_i_ elevation and manganese ion (Mn^2+^) quenching was measured in striatal cells loaded with the ratiometric Ca^2+^ sensitive dye Fura-2 LR/AM. The dye-loading methods used were as previously described (Osanai et al., 2006; Tamura et al., 2014). In brief, the corticostriatal slice was placed in a small plastic chamber containing 100 μl ACSF with 20 μM Fura-2 LR/AM, 1 μM sulforhodamine 101 (Sigma), and 0.02% Cremophor EL (Sigma). The dish was incubated at 35°C for 45 min in the small chamber, and then washed with 100 μl ACSF at 35°C for 15 min. To ensure that the [Ca^2+^]_i_ change was attributed to a neuronal event, sulforhodamine 101-positive cells, corresponding to astrocytes, were excluded (Nimmerjahn et al., 2004). After dye-loading, the slice was transferred to a continuously superfused (2–2.5 ml/min) chamber, and the fluorescence was observed by an epifluorescence upright microscope (BX51WI, Olympus) equipped with a 20×, NA 1.0 water-immersion objective (Olympus). The Fura-2 LR-loaded slices were excited at wavelengths of 340 or 380 nm using a filter changer (Lambda DG-4, Sutter Instruments, Novato, CA, USA) equipped with excitation filters (26-nm bandpass filter for 340 nm wavelength and 14-nm bandpass filter for 380 nm wavelength, Semrock), and fluorescent signals at 510 nm were captured (F340 or F380) every 2 s with an EM-CCD camera (DU-885, Andor Technology, Belfast, UK). Ca^2+^ imaging was performed in the presence of 1 μM tetrodotoxin (TTX, Nacalai tesque, San Diego, CA, USA) to avoid Ca^2+^ elevations caused by the opening of voltage-gated Ca^2+^ channels due to action potentials. The experiments were performed at 30 ± 1°C.

We identified GFP-positive cells (i.e., GABAergic neurons) by observing green fluorescence excited at 488 nm (6-nm bandpass filter, Semrock) and quantified the average fluorescence (F340 and F380) within the region of interest (ROI) of these cells as a function of time. [Ca^2+^]_i_ elevations in a striatal cell were estimated by the fluorescence ratio (*R* = F340/F380) from each imaged cell. The criterion for identifying neurons with the slow Ca^2+^ oscillations was whether they had a frequency of occurrence of spontaneous Ca^2+^ elevation above 0.001 Hz. The total recording duration was more than 4,200 s. All equipment was controlled by iQ software (Andor Technology, Belfast, UK). The analyses of the imaging data were performed with ImageJ software (Schneider et al., 2012) and custom-made programs (Supplementary Material) written in MATLAB (MathWorks, Natick, MA, USA).

### Mn^2+^ Quench Experiment

Mn^2+^ can pass through opened Ca^2+^-permeable channels and quenches the Fura-2 LR fluorescence emission (Amano et al., 1997; Kikuta et al., 2015). Thus, to evaluate Ca^2+^ influx from the extracellular space, the rate of the quench by Mn^2+^ was quantified as Δ F/F at 380 nm (Uehara et al., 2002; Tu et al., 2009; Kikuta et al., 2015). To evaluate the influx rate of Mn^2+^, the time constant of quenching (τ_q_) after MnCl_2_ perfusion was calculated by fitting with the following equation:

$$\Delta F/F=A_{b}\text{exp}\{-\left(t+T\right)/\tau_{b}\}+A_{q}\text{exp}(-t/\tau_{q})+B_{b}+B_{q}$$

where *t* is the time from the start of MnCl_2_ administration, *T* is the time between the onset of recording and the start of MnCl_2_ administration, *A*_b_, *τ*_b_, and *B*_b_ are amplitude, time constant, and baseline level of the fluorescence bleaching, respectively, and *A*_q_, *τ*_q_, and *B*_q_ are amplitude, time constant and baseline level of the fluorescence quenching, respectively. The curve fitting was performed using SciPlot (M. Wesemann, 1991–95).

### Immunohistochemistry

Mice were anesthetized with sodium pentobarbital (50 mg/kg, intraperitoneal) and perfused transcardially with PBS, followed by fixation with 4% paraformaldehyde in 0.1 M phosphate buffer (pH 7.4). The sections (30 μm thick) were incubated with primary antibody for STIM1 (rabbit, 1:250, Alomone lab, Jerusalem, Israel), STIM2 (rabbit, 1:500, Alomone lab, Jerusalem, Israel), Orai1 (rabbit, 1:500, Alomone lab, Jerusalem, Israel), Orai2 (rabbit, 1:500, Alomone lab, Jerusalem, Israel), or Orai3 (rabbit, 1:500, ProSci, Poway, CA, USA), along with anti-GFP antibody (goat, 1:5,000, Frontier Institute, Hokkaido, Japan). These sections were incubated with species-specific secondary antibodies conjugated to Alexa488 (1:200, Invitrogen, Carlsbad, CA, USA) and Cy3 (1:200, Jackson ImmunoResearch, West Grove, PA, USA). Cellular nuclei were counterstained with DAPI (Sigma-Aldrich, St. Louis, MO, USA). Fluorescent images were visualized under a confocal laser-scanning microscope (A1R, Nikon, Tokyo, Japan) equipped with proper filter cube specifications.

For cell counts, three sections through the dorsolateral striatum along the rostrocaudal axis between −1.10 and −0.38 (mm) from the bregma were prepared from each mouse. The number of immunoreactive cells in the regions of interest (210 × 210 μm) was counted in a computer-assisted manner (Adobe Photoshop CC2018, San Jose, CA, USA), and the number of double-labeled cells was divided by that of the total GFP-positive cells in each section. The mean percentage obtained from the three sections was calculated. We used four mutant mice for quantification in each immunostaining condition.

### Statistics

Statistical analyses were performed using JMP Pro 11 (SAS Institute, Cary, NC, USA). Statistically significant differences (*p* < 0.05) were assessed by the Mann–Whitney *U* test. All data are presented as mean ± SEM unless stated otherwise.

### Drugs

All drugs were applied by perfusion. TTX (1 μM) was used to block action potentials. Thapsigargin (Nacalai Tesque; 2 μM) was used to deplete ER Ca^2+^ by blocking smooth ER Ca^2+^-ATPase. SKF96365 (Tocris; 10 μM) was used to inhibit SOCCs. All other chemicals were purchased from Nacalai Tesque except for immunohistochemical agents.

## Results

### SOCCs in Striatal GABAergic Neurons

**Figure 1:**
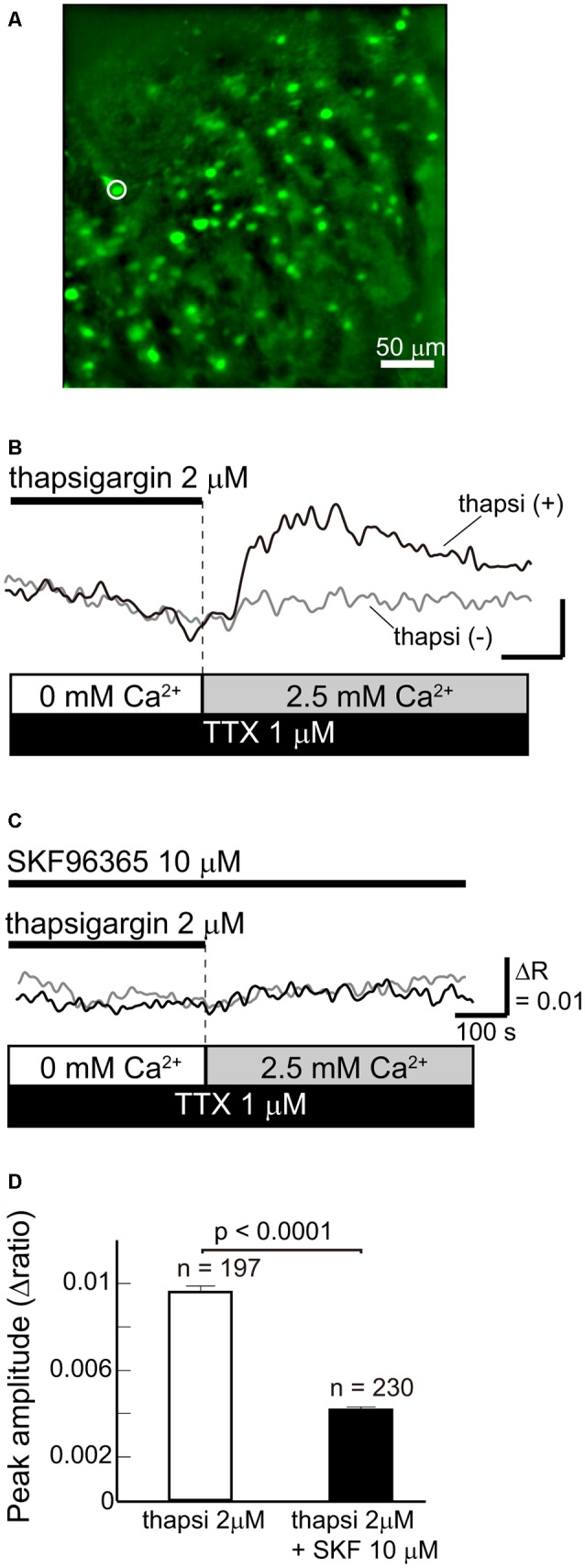
Functional store-operated calcium channels (SOCCs) existed in striatal GABAergic neurons. **(A)** Fluorescence image of a striatal slice from a GAD67-GFP mouse. Striatal GABAergic neurons were identified by GFP fluorescence (green) and ROIs were placed on the GFP-positive somata for quantifying fluorescence changes. Typical [Ca^2+^]_i_ time courses presented in **(B,C)** were obtained from the cell marked with a white circle on the fluorescence photo. **(B)** [Ca^2+^]_i_ elevation was induced by pretreatment with thapsigargin [black line, thapsi(+)] for more than 5 min in Ca^2+^-free conditions and subsequent perfusion of 2.5 mM Ca^2+^ (gray bar). The scale is the same as in **(C)**. **(C)** The [Ca^2+^]_i_ elevation induced by thapsigargin was blocked by 10 μM SKF96365. **(D)** The effect of SKF96365 on the peak amplitude of Ca^2+^ elevations induced by endoplasmic reticulum (ER) depletion.

To elucidate whether SOCCs are present in striatal GABAergic neurons, we performed fluorescence imaging with Fura-2 LR in acute slice preparations obtained from GAD67-GFP knock-in mice in which GFP was expressed in GABAergic neurons (Figure 1A). As more than 95% of neurons in the striatum are GABAergic projection neurons (Gerfen and Bolam, 2010), GFP-positive striatal cells in these mice were mostly projection neurons.

To deplete Ca^2+^ from the ER, Ca^2+^-free ACSF with 2 μM thapsigargin, an ER Ca^2+^-ATPase inhibitor, was perfused for more than 5 min. Subsequently, when the extracellular Ca^2+^ concentration increased to 2.5 mM following normal ACSF perfusion, GABAergic neurons exhibited remarkable elevations of [Ca^2+^]_i_ (Figures 1B,D). The [Ca^2+^]_i_ elevation was not observed when the ER was not depleted of Ca^2+^ by thapsigargin administration (Figure 1B). The [Ca^2+^]_i_ elevation induced by ER depletion was significantly reduced after the application of 10 μM SKF96365, a SOCC inhibitor (Baba et al., 2003; Bouron et al., 2004; Shideman et al., 2009; Figures 1C,D). The peak amplitudes of [Ca^2+^]_i_ elevation without and with SKF96365 were 0.0096 ± 0.0003 (*n* = 197 cells, three slices, three mice) and 0.0042 ± 0.0002 (*n* = 230 cells, three slices, two mice; *p* < 0.0001), respectively. These results indicate that SOCCs exist in striatal GABAergic neurons and exert some functional role.

### Involvement of SOCCs in Spontaneous Slow Ca^2+^ Oscillations

**Figure 2:**
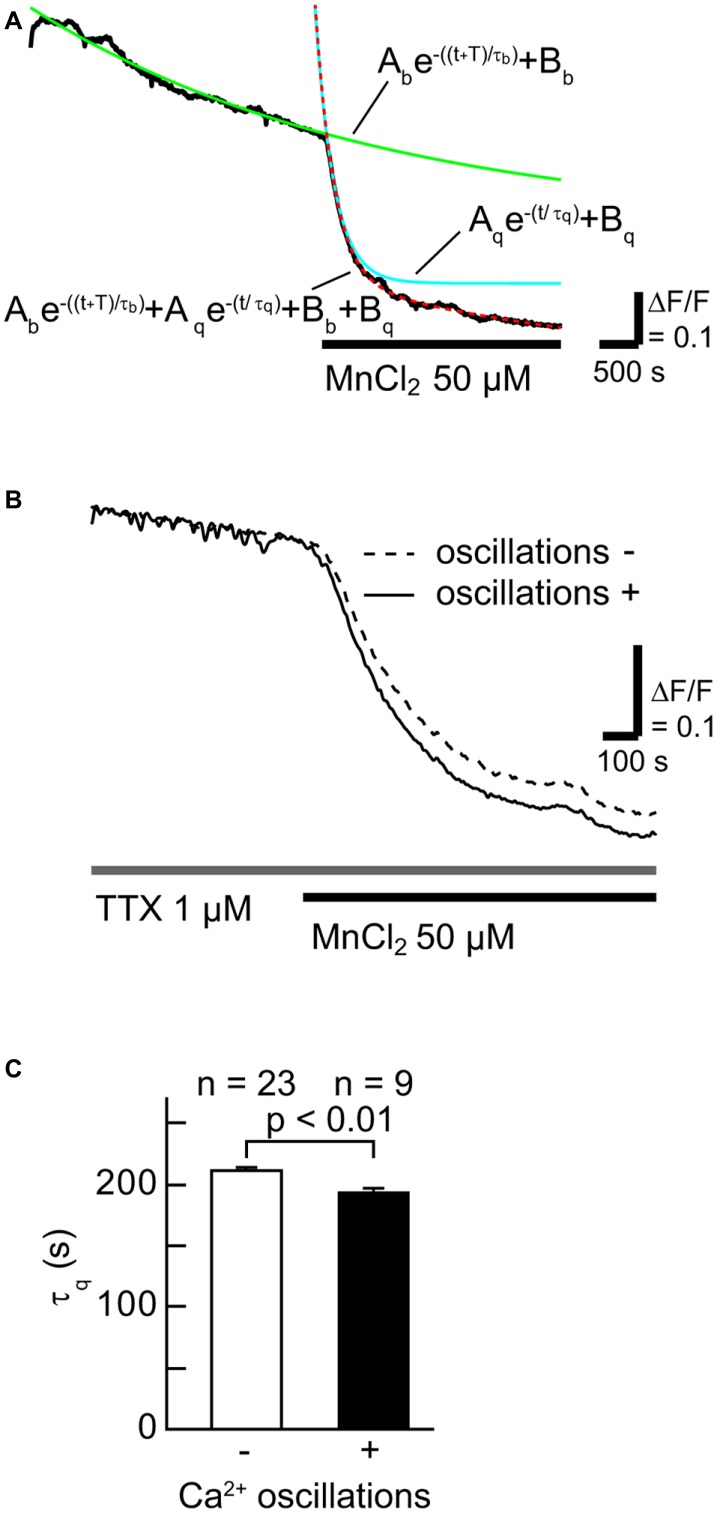
The rate of Ca^2+^ influx in neurons with slow Ca^2+^ oscillations was faster than in those without slow Ca^2+^ oscillations. **(A)** A typical time course of Mn^2+^ quench of Fura-2 LR fluorescence (black line). To evaluate the rate of Ca^2+^ channel opening, the fluorescence decay was fitted with the double-exponential function (dotted red line) consisting of a fluorescence bleaching component (green line) and a quenching component (blue line). **(B)** Typical time courses of Mn^2+^ quench in neurons with (solid line) and without (broken line) slow Ca^2+^ oscillations. **(C)** Summary data of the time constant of the quenching component (τ_q_) in neurons without (open) and with (filled) slow Ca^2+^ oscillations.

To examine whether SOCCs are responsible for maintaining high [Ca^2+^]_i_ in striatal GABAergic neurons exhibiting spontaneous slow Ca^2+^ oscillations, we first tested the contribution of Ca^2+^-influx from the extracellular space in striatal GABAergic neurons with and without slow Ca^2+^ oscillations by quantifying the time constant of fluorescence quench (Figures 2A,B). More Mn^2+^ entry from the extracellular space caused faster fluorescence quench (Amano et al., 1997; Uehara et al., 2002; Tu et al., 2009). The Fura-2 LR fluorescence quench was observed after MnCl_2_ (50 μM) administration in the neurons with slow Ca^2+^ oscillations (Figure 2B). The time constants τ_q_ of fluorescence quench were 211 ± 4 s (*n* = 23) in the neurons without slow Ca^2+^ oscillations and 193 ± 4 s (*n* = 9) in those with these oscillations (Figure 2C, *p* < 0.01). These results suggest that more Ca^2+^ enters those striatal neurons with spontaneous slow Ca^2+^ oscillations than those without the oscillations.

**Figure 3:**
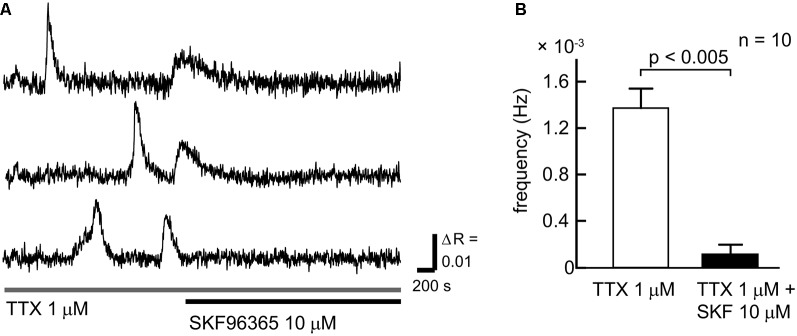
SOCCs were involved in the spontaneous Ca^2+^ oscillations. **(A)** Typical time courses of spontaneous slow Ca^2+^ oscillations during the administration of 1 μM TTX (gray bar) and 10 μM SKF96365 (black bar). Spontaneous Ca^2+^ elevations were not present following the application of SKF96365. **(B)** Frequencies of [Ca^2+^]_i_ elevations in the absence (open bar) or presence (solid bar) of SKF96365. The frequency of spontaneous Ca^2+^ oscillations was significantly reduced by SKF96365 administration.

To clarify the contribution of SOCCs to spontaneous slow Ca^2+^ oscillations we blocked the channels with SKF96365 (Figure 3). To avoid [Ca^2+^]_i_ elevation due to action potentials, TTX was applied during the experiments. The slow Ca^2+^ oscillations nearly disappeared in the presence of SKF96365 (Figures 3A,B). The frequency of the oscillations in the control condition was 1.27 ± 0.16 × 10^−3^ Hz, whereas in the presence of SKF96365 the frequency was 0.109 ± 0.078 × 10^−3^ Hz (*n* = 10 cells, five slices, four mice, *p* < 0.005). Thus, SOCCs clearly contribute to the occurrence of spontaneous slow Ca^2+^ oscillations in striatal GABAergic neurons.

### Molecular Components of SOCE in Striatal GABAergic Neurons

**Figure 4:**
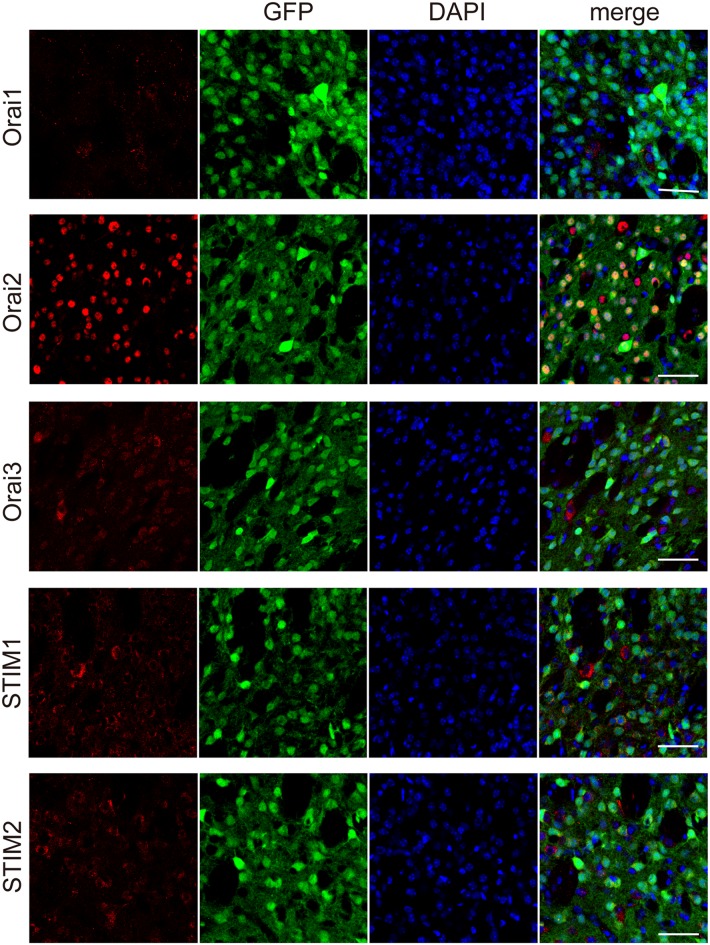
Each of the molecular components of SOCE expressed at different levels in the striatal GFP-positive cells. Confocal microscopy images of the sections through the dorsal striatum obtained from GAD67-GFP mice, which were stained for Orai1, Orai 2, Orai3, STIM1, or STIM2 (red), along with GFP (green). Blue staining represents DAPI. Scale bar: 50 μm.

The major molecular components of the store-operated Ca^2+^ entry (SOCE) are STIM (STIM1 and 2) and Orai (Orai1, 2, and 3; Kraft, 2015; Moccia et al., 2015). Through immunohistochemical analyses, we examined the expression of Orai1, 2, and 3, the pore-forming subunit of the “Ca^2+^-release-activated Ca^2+^ (CRAC)” channels (Kraft, 2015; Moccia et al., 2015), in striatal GABAergic neurons. In striatal GABAergic neurons, strong expression of Orai2 and moderate expression of Orai3 were observed, while Orai1 expression was detected only in a few cells (Figure 4). The percentages of the Orai1-, Orai2-, and Orai3-positive cells relative to the total GFP-positive cells were 4.7 ± 2.3, 97.8 ± 0.9, and 62.5 ± 1.6, respectively (%, *n* = 4 mice). We also examined the expression of STIM1 and 2, the ER Ca^2+^ sensor activating CRAC channels on the plasma membrane (Kraft, 2015; Moccia et al., 2015). Both of STIM1 and 2 were expressed in striatal GABAergic neurons (Figure 4). The percentages of the STIM1- and STIM2-positive cells relative to the total GFP-positive cells were 76.1 ± 3.8 and 75.5 ± 3.3, respectively (%, *n* = 4 mice). These results suggest that in striatal GABAergic neurons, Orai2 is a major molecular component of SOCCs, and both STIM1 and STIM2 contribute to the activation of this Orai protein.

## Discussion

In the present study, we demonstrated that striatal GABAergic neurons exhibit SOCE (see Figure 1), and that blocking this Ca^2+^entry greatly reduces the frequency of spontaneous slow Ca^2+^ oscillations in these neurons (see Figure 3). Wu et al. (2016) observed elevations in [Ca^2+^]_i_ after depleting Ca^2+^ from the ER, which is considered to be ascribed to SOCE (Majewski and Kuznicki, 2015), in corticostriatal co-culture preparations. The time course of this Ca^2+^ elevation was similar to that observed in our work. The Ca^2+^ elevation after the ER Ca^2+^ depletion in striatal GABAergic neurons was almost completely blocked by SKF96365, an inhibitor of SOCCs (see Figure 1). Taken together, these findings indicate that functional SOCCs exist on the plasma membrane of striatal GABAergic neurons.

The major molecular components of the SOCE pathway are STIM (STIM1 and 2) and Orai (Orai1, 2, and 3) proteins (Kraft, 2015; Moccia et al., 2015; Secondo et al., 2018; Wegierski and Kuznicki, 2018). Therefore, we investigated which molecules were responsible for the SOCE in striatal GABAergic neurons. In the immunohistochemical study, STIM1 and 2 were expressed equally in striatal GABAergic neurons (see Figure 4). This observation is consistent with previous data showing that both STIM1 and 2 are widely expressed in the rodent brain (Kraft, 2015; Moccia et al., 2015; Wegierski and Kuznicki, 2018). On the other hand, Orai2 was strongly expressed in striatal GABAergic neurons, whereas Orai1 was expressed in only a few GABAergic neurons (see Figure 4). Although Orai1 is known to be expressed extensively in the rodent brain (Moccia et al., 2015), its expression level in striatal GABAergic neurons, which is the principal component of striatal neurons, has not yet been reported. Thus, Orai2 but not Orai1 may be a major pore-forming protein of SOCE in striatal GABAergic neurons. In addition, Orai3 was also expressed in striatal GABAergic neurons, albeit its immunoreactivity was not so intense as that for Orai2 (see Figure 4). Further investigations are needed to elucidate a molecular substrate for SOCE in striatal GABAergic neurons.

We previously observed spontaneous slow Ca^2+^ oscillations in striatal neurons and astrocytes (Osanai et al., 2006; Tamura et al., 2014) that were blocked by mGluR5 or IP3R inhibition (Tamura et al., 2014). In addition, the mGluR5-dependent sustained Ca^2+^ elevation was strongly blocked by Zn^2+^, which is thought to be a SOCC blocker (Uehara et al., 2002), in rat cortical neurons and glia (Prothero et al., 1998). It was also shown that group I mGluRs including mGluR5 were related to SOCE in midbrain dopaminergic neurons (Tozzi et al., 2003), and that the application of the SOCC blocker 2-aminoethoxy-diphenyl borane (2-APB) totally abolished the response to DHPG (group I mGluR agonist) in about half of midbrain auditory neurons (Martinez-Galan et al., 2012). Therefore, activation of mGluR5 may lead to SOCE. In the present experiments, we took advantage of the fact that Mn^2+^ ions readily enter cells through Ca^2+^-conducting channels (Uehara et al., 2002; Tu et al., 2009; Chen et al., 2015) and quench Fura-2 LR fluorescence to verify that SOCCs contribute to the spontaneous slow Ca^2+^ oscillations that depend on the mGluR5-IP3R signal transduction cascade. The fluorescence was quenched faster in the neurons with the slow Ca^2+^ oscillations than in those without these oscillations (see Figure 2), indicating that the amount of extracellular Ca^2+^ influx in the neurons with the slow Ca^2+^ oscillations was larger than in those without the oscillations, and thereby suggesting that more SOCCs open in striatal neurons exhibiting spontaneous slow Ca^2+^ oscillations.

In our previous observations, the maximum duration of a single event of the slow Ca^2+^ oscillations was approximately 300 s (Osanai et al., 2006; Tamura et al., 2014). However, how such a long-duration Ca^2+^ elevation is maintained remains to be resolved. If SOCCs contribute to maintain high Ca^2+^ concentrations in single oscillatory events, the duration of the Ca^2+^ elevation must be shortened by SOCC blockade. However, the frequency of the slow Ca^2+^ oscillations in striatal GABAergic neurons was dramatically reduced (see Figure 3). This, together with the fact that Ca^2+^ flows into the cell through SOCCs when ER stores are depleted, implies that SOCCs are responsible for the initiation of Ca^2+^ elevation in spontaneous slow Ca^2+^ oscillations, and SOCCs have a potential for maintaining the long-duration Ca^2+^ elevation.

SOCCs have been most thoroughly studied in non-neuronal cells. Although their physiological and pathological roles have been argued, accumulated evidence suggests that dysregulation of SOCE triggers perturbation of intracellular Ca^2+^ signaling that participates in key physiological functions, including synaptic plasticity, axonal growth, and synaptic formation, and in the pathogenesis of neurodegenerative diseases, including Alzheimer’s disease (AD), Huntington’s disease (HD), and Parkinson’s disease (Bardo et al., 2006; Majewski and Kuznicki, 2015; Secondo et al., 2018; Wegierski and Kuznicki, 2018). Moreover, abnormal mGluR5-IP3R signal transduction is thought to be involved in SOCE related to perturbation of intracellular Ca^2+^ signaling in AD and HD (Amano et al., 1997; Secondo et al., 2018). Wu et al. (2016) reported that the increase in steady-state IP3R activity resulted in overactivation of SOCE in cultured medium spiny projection neurons (MSNs) of the striatum in an HD mouse model characterized by age-dependent dendritic spine loss in MSNs, and that inhibition of SOCE rescued such a spine loss. These results indicate that the striatal SOCCs may contribute to neuronal degeneration in an HD model. As the present study was conducted in normal mice, we cannot conclude that spontaneous slow Ca^2+^ oscillations generate abnormal Ca^2+^ signals induced by overactivation of SOCCs in the striatum.

Intracellular Ca^2+^ can modulate protein functions, gene expressions, and morphological changes in cellular processes, thus leading to modified functions of neurons and neuronal circuits (Berridge et al., 2000). The SOCCs have also been implicated in synaptic plasticity (Baba et al., 2003; Bardo et al., 2006; González-Sánchez et al., 2017; Korkotian et al., 2017). Therefore, the SOCCs contributing to the occurrence and the maintenance of spontaneous slow Ca^2+^ oscillations in striatal GABAergic neurons might regulate related network functions. Further investigations are needed to elucidate the role of SOCCs in striatal GABAergic neurons.

## Data Availability Statement

All datasets generated for this study are included in the article/Supplementary Material.

## Ethics Statement

The Tohoku University Committee for Animal Experiments approved all animal experiments, and the experiments were performed in accordance with the Guidelines for Animal Experiments and Related Activities of Tohoku University, the guiding principles of the Physiological Society of Japan and the National Institutes of Health (NIH), USA.

## Author Contributions

SK, YI, TK, KK, and MO conceived and performed experiments. SK, YI, and MO analyzed data. YY provided the GAD67-GFP mice and information for maintaining them. SK, YI, YY, MT, and MO wrote the manuscript.

## Conflict of Interest

The authors declare that the research was conducted in the absence of any commercial or financial relationships that could be construed as a potential conflict of interest.
